# Endoscopic Management of a Tubulovillous Adenoma Within a Diverticulum: Report of a Case and Review of Literature

**DOI:** 10.7759/cureus.8668

**Published:** 2020-06-17

**Authors:** Fadi Hawa, Alsadiq Al Hillan, Andrew T Catanzaro, Joseph A Tworek, Naresh T Gunaratnam

**Affiliations:** 1 Internal Medicine, St. Joseph Mercy Ann Arbor Hospital, Ann Arbor, USA; 2 Internal Medicine, Jersey Shore University Medical Center, Neptune City, USA; 3 Gastroenterology and Hepatology, St. Joseph Mercy Ann Arbor Hospital, Ann Arbor, USA; 4 Pathology, St. Joseph Mercy Ann Arbor Hospital, Ann Arbor, USA

**Keywords:** colon cancer and colon polyps, tubulovillous adenoma, colon cancer prevention, endoscopic approach, endoscopic mucosal resection, endoscopic submucosal dissection, colorectal neoplasia, banding without resection, colonic diverticulum, case report

## Abstract

Adenomas or adenocarcinomas located within a colonic diverticulum are considered a rare phenomenon that has been described in the literature. These lesions are technically difficult to manage endoscopically and usually require surgical intervention for removal. There is also an increased risk of perforation upon endoscopic resection owing to the lack of a muscular layer within the diverticulum.

We report a case and include a literature review to evaluate different endoscopic techniques and propose the most effective for management of adenomas within a diverticulum. This technique is potentially comprised of employing a combined approach using a suction banding device, an over-the-scope clip (OTSC; Ovesco Endoscopy AG, Tübingen, Germany) , and hyperthemic snare to successfully remove the polyp, ensure tissue retrieval, and reduce risk of iatrogenic colonic perforation.

## Introduction

Adenomatous polyps are the most prevalent neoplastic polyps in the colon and comprise approximately two-thirds of all colonic polyps [1]. Adenomas or adenocarcinomas located within a diverticulum are a rare phenomenon that has been described in the literature [2-5]. These lesions pose a technical challenge for endoscopic management given their difficult location inside the diverticulum and the greater risk of perforation upon endoscopic resection owing to the lack of the muscular layer within the diverticulum [4-6]. Therefore, surgery is considered the standard treatment for colorectal neoplasia (CRN) associated with a diverticulum [7]. 

Endoscopic techniques for resection of tumors arising in a diverticulum have been proposed in the literature as less-invasive approaches [7-9]. However, the evidence supporting this approach remains modest and controversial. In our case study, we evaluate an endoscopic technique for the management of a tubulovillous adenoma within a diverticulum with review of the literature. We aim to explore an effective, potentially safe, and feasible endoscopic approach to manage this phenomenon.

## Case presentation

**Figure 1:**
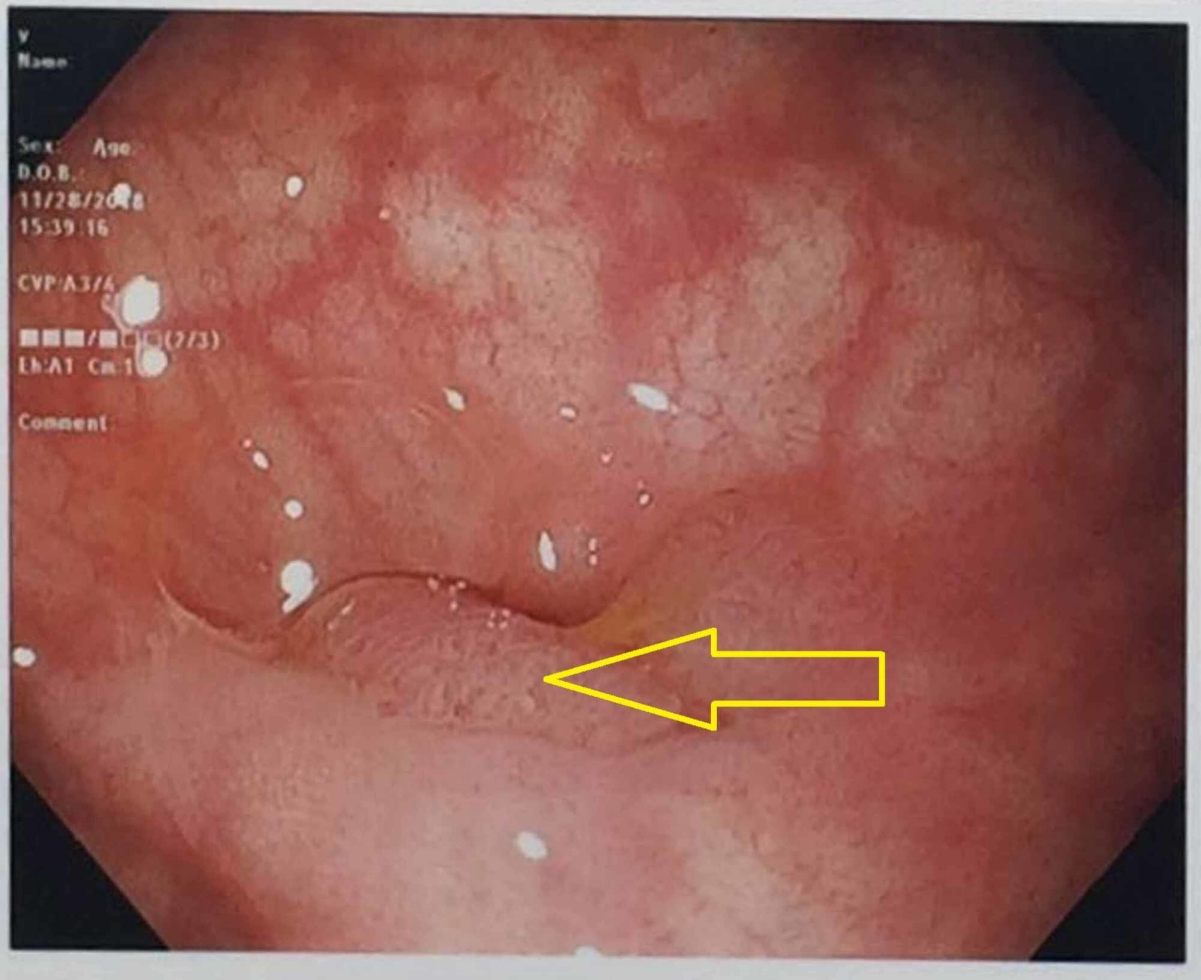
Sigmoid colon pedunculated polyp within a diverticulum (yellow arrow)

A 74-year-old male with a past medical history of hypertension, hyperlipidemia, and coronary artery disease was referred by his primary care physician for colon cancer screening on a background history of loss of appetite and 20 pounds weight loss without prior colonoscopies. On presentation, vital signs were within normal limits and physical examination was unremarkable. Subsequently, the patient underwent index screening colonoscopy which identified a 15-mm pedunculated polyp within a sigmoid colon diverticulum. Despite multiple attempts, the polyp could not be lifted with forceps or safely snared as the majority of the polyp was within the diverticulum. A superficial biopsy of the polyp confirmed a tubulovillous adenoma. Accordingly, the site was tattooed and the patient was referred for endoscopic mucosal resection (EMR). A follow-up flexible sigmoidoscopy using a gastroscope (Olympus GIF-H190, Tokyo, Japan) identified a prolapsing pedunculated polyp located within a diverticulum at 25 cm from the anal verge (Figure 1). 

**Figure 2:**
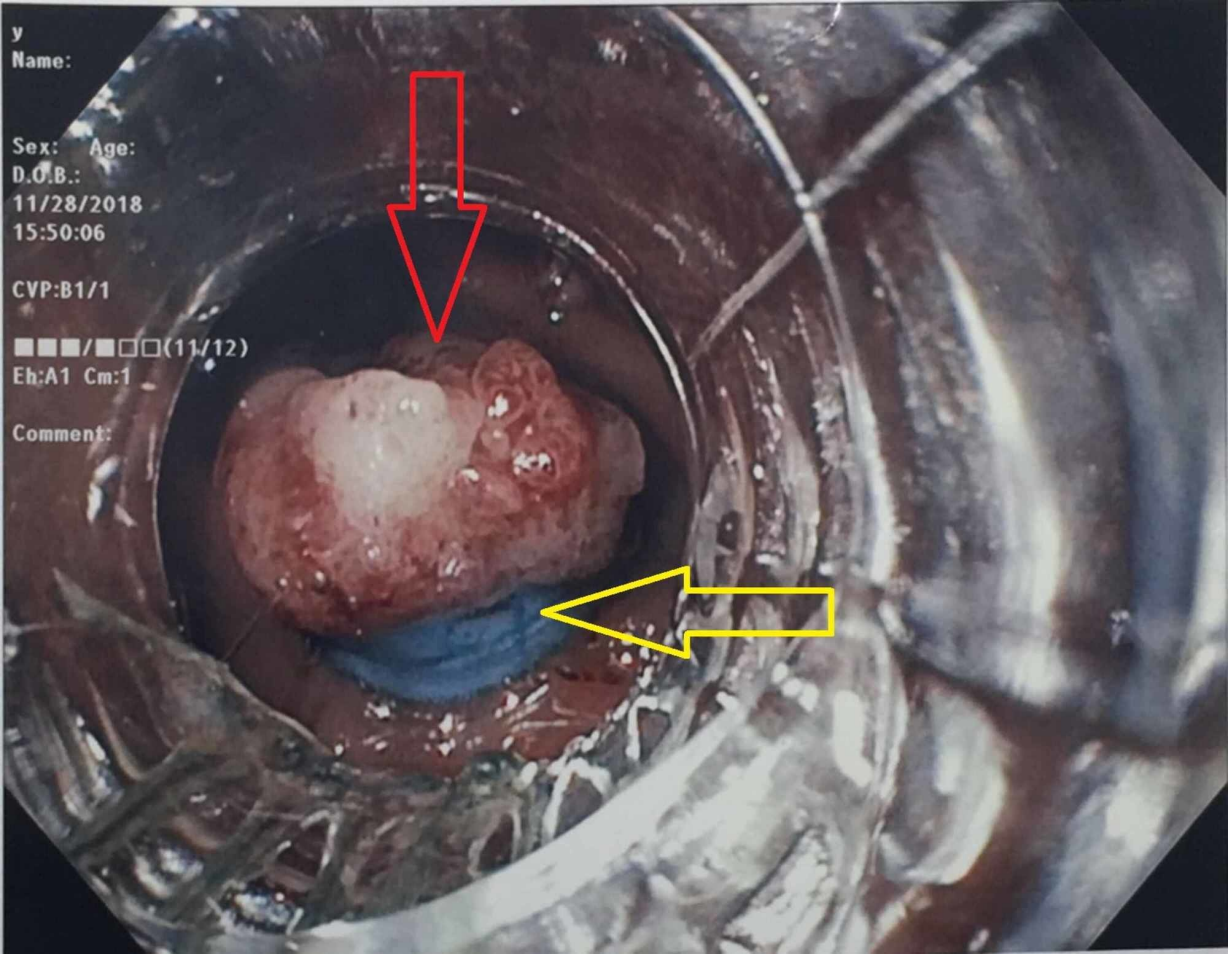
Exposed polyp (red arrow) after placement of two elastic bands (yellow arrow)

Initially, a 10-mm stiff snare was tried to expose the polyp, but was unsuccessful. This was followed by submucosal injection of saline at the base of the polyp in hopes to lift the lesion which was also unsuccessful. Therefore, after multiple failed attempts to lift the lesion, the endoscopist elected to use a variceal banding kit (Speedband Superview Super, Boston Scientific, Natick, MA, USA) which was positioned above the apex of the polyp. Suction was applied which successfully pulled the entire polyp within the band ligator. Two bands were deployed across the base of the polyp which exposed the entire lesion (Figure 2). 

**Figure 3:**
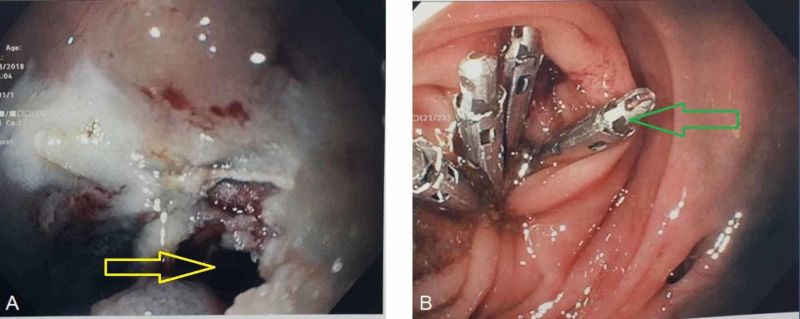
(A) Endoscopic resection complicated by 5-mm colonic perforation (yellow arrow). (B) Defect successfully closed with placement of four endoclips (green arrow)

Hyperthermic snare resection using a 10-mm stiff snare and the ERBE generator (forced coagulation current/20 watts) was performed above the elastic bands in hopes to reduce the risk for iatrogenic perforation. Complete resection with tissue retrieval was done; however, inspection of the resection site demonstrated a 5-mm perforation which was immediately closed using four endoclips (HX-610-090; Olympus Medical Systems Corp., Tokyo, Japan) (Figure 3). 

**Figure 4:**
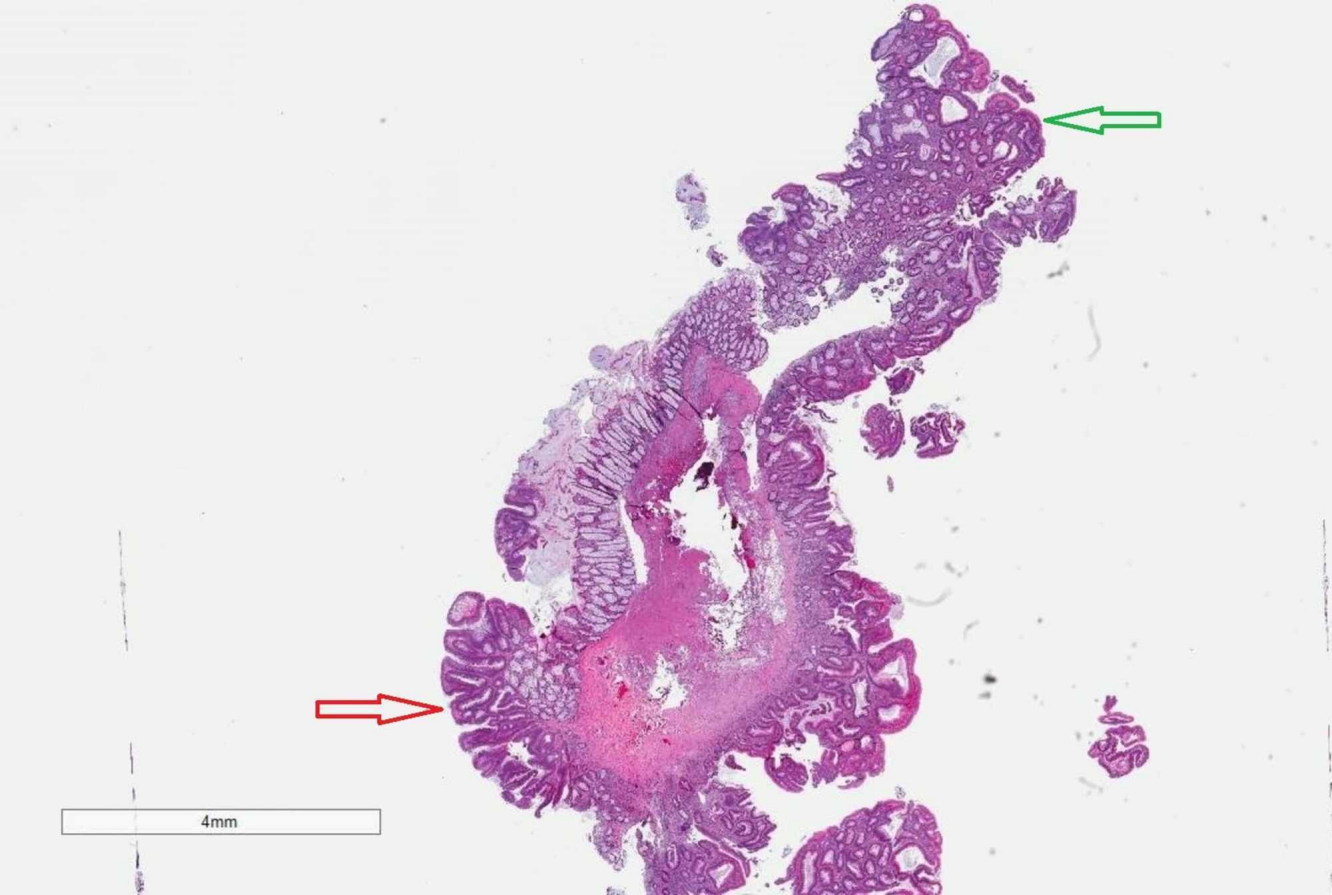
Tubulovillus adenoma histopathology. A polyp with a broad-based central stalk containing smooth muscle of muscularis mucosa. The stalk is lined in the lower left (red arrow) by distinct papillary projections imparting a villous configuration and in the top right (green arrow), it is lined by a flattened proliferation imparting a non-villous configuration. Both areas of the tubulovillus adenoma contain cells with elongated nuclei that stratify toward the surface of the polyp. (Hematoxylin-eosin, original magnification x6)

The patient was admitted overnight for observation and was discharged the following day without any complications. Pathology confirmed a 15-mm tubulovillous adenoma without dysplasia (Figure 4). The patient has been followed up to 15 months post-procedure without any signs of delayed complications. 

## Discussion

CRN confined to the mucosal layer can be successfully managed by endoscopic treatments, such as EMR or endoscopic submucosal dissection (ESD) [10]. However, polyps located within a diverticulum are technically challenging to resect given difficulties of isolating the lesion. Techniques like submucosal injection and positioning snares against the diverticulum may be effective in exposing the polyp, but failed in our case. Suction of the polyp using a band ligator and deployment of elastic bands across the base of the polyp was effective in isolating the polyp and preventing it from prolapsing back into the diverticulum. The resection however was complicated by a perforation most likely due to the absence of the muscular layer in the wall of the diverticulum. 

Generally, EMR and endoscopic resection of banded tissues are considered challenging in cases of diverticula-associated CRN due to the increased risk of perforation secondary to the lack of a muscular layer [4,11,12]. On the other hand, ESD safety and feasibility has been demonstrated for these particular lesions in a more recent retrospective case series by Jimenez-Garcia et al. with better en block resection rates for tumors located near a diverticulum than those extending into the diverticulum [7]. 

Interestingly, allowing the banded polyp to slough off secondary to induced ischemia by the elastic band rather than using electrocautery may have been effective in removing the polyp. Ishii and Fujita reported using band ligation without resection of a colonic polyp extending into a diverticulum, which resulted in resolution of the diverticulum and the associated polyp on follow-up colonoscopy performed after two months [11]. Furthermore, Carmo et al. utilized a similar approach for a 6-mm sessile polyp within a sigmoid colon diverticulum and repeat endoscopic evaluation in two weeks revealed no evidence of a residual polyp [13]. The main drawback to this technique is the lack of tissue retrieval and histopathological evaluation which confines it only to diminutive lesions up to 10 mm in size given their negligible risk (0.2%) of submucosal invasion [9,11,13].

Other endoscopic approaches for colonic polyps within a diverticulum have been reported in the literature. Valli et al. described a 10-mm polyp within an ascending colon diverticulum [14]. The polyp was removed using a full-thickness resection device (FTRD) [15]. A therapeutic colonoscope was initially fitted with the FTRD device consisting of an over-the-scope clip (OTSC; Ovesco Endoscopy AG, Tübingen, Germany) mounted onto a transparent cap and hyperthermic snare within. Both the adenoma and the diverticulum were suctioned into the plastic cap of the FTRD. OTSC was then deployed followed by full-thickness colon resection above the OTSC using the standard hyperthermic snare. Histopathological examination revealed tubular adenoma. No early or late post-procedure complications were reported. Shakhatreh et al. described a 10-mm sessile polyp within a transverse colon diverticulum [16]. During colonoscopy, the polyp and the surrounding diverticulum were suctioned into the cap of a banding device with deployment of one band at the polyp’s neck. Next, an OTSC was deployed around the banded polyp under the rubber band. Finally, the polyp was removed with hypethermic snare performed above the clip. Histopathological examination confirmed a tubular adenoma. No early or late post-procedure complications were reported. All reported cases including ours required mechanical isolation of the polyp with the use of a suction banding device or an FTRD [14,16,17]. Both techniques were equally effective in extracting and isolating the polyp out of the diverticulum. However, using hot snare polypectomy for a banded polyp will likely result in a perforation as witnessed in our patient, which will require immediate closure with endoscopic clips to avoid complications, such as peritonitis. Therefore, such cases should be performed by experienced endoscopists and a rather cautious approach needs to be employed by placing an OTSC prior to endoscopic resection in order to minimize the risk of iatrogenic colonic perforation [18,19]. 

## Conclusions

Colonic polyps within a diverticulum are uncommon, yet technically challenging to resect. However, they can be successfully removed by using a suction banding device or FTRD to isolate the polyp. If endoscopic resection is pursued, an OTSC should be used prophylactically prior to resection in order to prevent an iatrogenic perforation. Allowing the banded polyp to slough off secondary to tissue ischemia induced by the band is possibly the safest and the most technically feasible method. However, it should be used in selected patients only when tissue retrieval is not felt to impact patient's outcomes and lesion size does not exceed 10 mm. 
